# Mechanical Dentistry Forty Years Ago

**Published:** 1909-05-15

**Authors:** Edwin D. Downs

**Affiliations:** Owego, N. Y.


### MECHANICAL DENTISTRY FORTY YEARS AGO.

BY EDWIN D. DOWNS. D.D.S., OWEGO, N. Y.

Read before Sixth District Dental Society, at Elmira, N. Y., October 3, 1908.

If I were to confine myself to the title of my paper, I should simply say to you that "Mechanical Dentistry Forty Years ago," consisted of rubber plate work,—and sit down.

It has been suggested to me that a great many "Continuous Gum" plates had been made at the time of the organization of this society, but as my memory goes back to only nine years later, and as only a few had ever come under my personal observation, I have my doubt of it. More than that I have become convinced by my reading in the preparation for this paper, that continuous gum work never came into general use. It was prominent in discussions then and later and I became so much interested in the subject that I learned the process; my deposit plate at college was "continuous gum."

Dr. D. D. Smith, who wrote the article on this form of work for the "American System of Dentistry" and who is a friend and advocate of the work, says in his article: "Whatever favor it may have gained from the time of its introduction, 1851 to 1860 the time of the general adoption of vulcanite, it as quickly lost out before its cheaper and more easily constructed rival. After the full introduction of vulcanite, interest in metal work—Gold, silver or platinum—rapidly declined. * * * * * * * Metal plates were esteemed so unimportant that students were uninstructed in any of their forms, until a generation of students came upon the stage literally incapable of mounting a tooth on a gold or silver case, and without even having as much as seen a set of continuous gum Work."

Harris says of the progress of vulcanite: "* * * * * * that in 1858 not more than 300 dentists made any use of it. In 1863 nearly 3,000 used it; in 1870 not more than 300 did not use it." Therefore I think it is proper for me to say that my subject, as described by the title of my paper, is covered when I state mechanical dentistry forty years ago was rubber plate work. Of course there were men doing more or less metal work, for when this and the other district societies were organized in 1868, nearly all the organizzers must have been trained in metal work; but also at that time mechanical dentistry must have been near the low water mark, for—here was the easy work the kind whose price made it possible for an enormous number of people to have dental service, heretofore debarred from it, opening up a large field for exploitation. Do you doubt the result?

The advent of vulcanite was a revolution—its general adoption seemingly a calamity. Theretofore a man had to have a considerable amount of mechanical skill to practice our profession. Mechanical skill usually means a well developed brain—but not always a sweet temper,—and I think it fair to say that previous to the advent of rubber, the majority of dentists were mentally a superior lot of men. It would be interesting to trace their descendants: I think they would be found giving a good account of themselves, all doing honor to the old plate swager.

But with rubber came the flood—three months in a dental office and the boy fresh from the farm was a full fledged dentist; and this amount of tutelage to the boy from the carpenter's bench gave him the reputation of great skill, especially if he decorated his office with a few examples of his skill in carpentry. Was he not "a real ingenious" and "a good mechanic?"

Of course these men attempted tooth filling, and no dentist who has come into practice within ten, or even a few more, years, knows how badly a tooth can be filled. The real "red rubber dentist" has passed into the Great Beyond; but what they encouraged, and where they did their great harm, was inducing people to have out whole mouths full of teeth, most of which could have been saved. Many good dentists would not admit to their confreres that they knew much about rubber work—they left that to their mechanical man,—and all the talk was of divorce of the office and laboratory. We heard of the eye and ear specialists in medicine, and of all its other specialities, and why not specialists in the different branches of dentistry.

But out of the dark groweth the dawn. Both respectable dentists and intelligent people grew sick of "the things" that not only called themselves dentists, but wrote doctor ahead of their names whenever and wherever they found opportunity; out of it all came our dental laws—and we stopped to take breath.

But the red rubber and not much else was still with us, and the talk of divorce of office and laboratory still continued.

Perhaps I ought to mention that in the meantime a supposedly higher form of rubber work had appeared; that is, a gold plate with the teeth mounted with rubber, but none too many were sufficiently skilled to do good swageing, and we continued under the dominion of plain vulcanite.

Now do not misunderstand me; I am not belittling rubber plate work; it has been an inestimable blessing to humanity, in its cheapness, in its adaptability. It has not only saved many of the poor from the pangs of dyspepsia, but it reaches cases almost beyond the adaptability of metal, and has survived through its merits. But its first effect on dentistry was apparently disastrous; it was like a flood before the dykes had been finished. The competent and busy operator did plate work merely to fill his odd time and feed his operating room; he paid but little attention to mechanics; the laboratory was in the hands of a mechanical dentist or more likely a student. The dentist talked divorce of operating room and laboratory, but in the meantime crown and bridge work were growing in importance, and the two rooms began to look towards reconciliation; for this work was both operative and mechanical, and in its early days at least, the work went from one room to the other quite frequently during its making. No longer could a man be found who denied knowledge of his laboratory or admitted his inability to make all gold crowns, and to-day most of us like to talk as wisely of laboratory procedure as of the operating room, for every year seems to weld them closer. Porcelain and gold inlays? Are they progress of the operative or mechanical side? You give a different shape to your cavity walls, you make a new use of the cement you long have had, but the filling is constructed in the laboratory.

We talk wisely of the survival of the fittest. Rubber must be mighty fit for dental plates for human mouths, for it has survived a lot of abuse and hammering. The discussions over it were hot and furious. We were told in dental societies discussions how harmful it was to health. Celluloid was introduced and extensively used. Those who used it, and did not use rubber, advocated its merits loudly until the license fee on rubber was no longer demanded, then celluloid faded from dental practice.

As I write I wonder if I ought to say something of the vulcanite patent; certainly it was an important factcr in mechanical dentistry during a period between thirty and forty years ago. Whether it was lack of industry or not, I do not know, but I have been unable to find its history in books in my possession. Of course much of it is in the journals, but I did not have patience to search them.

Those who owned the rubber patent introduced a license system. The price of a year's license in Owego was fifty dollars, but if you paid the license in the early part of January, for a year in advance, it was thirty-five dollars. For a young man just starting and only getting an occasional plate to make, this was a rather serious matter.

The patent seemed easy to evade and many tried it. It seemed a matter between you and your patient. But the owners of the patent were equal to the emergency. You went along successfully for a time, then a collector appeared.

He was bright and up to date, and very affable; asked you how many plates you had made of vulcanite, and advised you to take out a license. You denied the rubber, showed him your celluloid apparatus (if you had one) and said it was giving your patients universal satisfaction, and he said "good bye," and you congratulated yourself on having fooled him. A few weeks later he was back, saying he understood you had made that nice set on rubber that Mrs. Brown was wearing; you went up in the air—so did he. Perhaps, after further excited talk, you settled. Perhaps you chased him out with a plaster knife—it was all a matter of temperament or monetary resources. It was said, the collectors had been kicked from offices so often that they could tell when a boot hit them, whether it was pegged or sewed, patent or ordinary leather; but this ability to diagnose boots has been credited to other vocations, and I think we may be permitted to doubt it. If you weakened and settled, you were duly happy, and naturally (even if meanly), perhaps began looking up evidence against some competitor who had not been caught. If instead of settling, you chased the collector out, or put up such a bluff that he went away apparently satisfied, you pretended to be happy and easy. But soon you were cited before a United States Court to show cause why you should not be enjoined from making plates on vulcanite. You had refused to take a license, you had no case, and the injunction issued. No damages for the past had been asked of you, and you found yourself plodding along in your office much the same as usual, except that you were growing red headed over business the other fellow was getting; you thought you could be careful enough not to be caught; you tried it, and found yourself in jail for contempt of court. Oh! it was a pretty system, and the hatred of the dentist for Josiah Bacon and his assistants was unutterable. But the end came; Josiah Bacon was found shot to death in his room in the Palace Hotel, San Francisco—shot in broad daylight. For many hours (more than twenty-four), not a clue to his murderer. Then a San Francisco dentist walked into a police station and gave himself up.

On the stand he told his story, and the story of vulcanite. He was convicted in a minor degree and sentenced to ten years imprisonment. A woman became interested, applied to dentists and dental societies to sign petitions for his pardon, and he was pardoned. I forget how long he served, but the more errors I make in this history, the better show will some of the older ones have in further discussion.

Indirectly, at least, I think I have compared the past with the present in speaking of inlays, etc. Certainly I do not propose to bore you further with descriptions of methods and processes that are part of your daily life.

I have taken the liberty of saying something of the rubber patent, thinking that the present generation might have little or no knowledge of that period. I have not tried to be exact or specific.

All our papers at this meeting are more or less historical. It is nice to sit around our camp fire, and recall the comrades and the things that are gone, but the chief value of history is in its application. The history of rubber teaches us that the thing despised and condemned is sometimes not only the thing that survives, but may carry with it blessings not predictable.

Out of it all let us learn that there can be no divorce of the operating room and the laboratory. Let us not scorn any branch of our vocation. Let us be scholars, but we *must* be mechanics. I entered dentistry under the reign of rubber, and at that period when a goodly number professed to despise the laboratory, and would have divided dentistry into two social classes, the operator and the mechanic—I was infected with the sentiment prevailing—it was a false sentiment and should never be tolerated amongst us again.

The ingenious mechanician who does not neglect study and science is the man to become a dentist.

Orthodontia alone offers untold opportunities for the ingenious craftsman, the man who can *do* things. Ready made apparatus you can buy, and it is good, but it is the mind of the engineer that must apply it.

The future of the laboratory is bound up, one and indissoluble with the future of dentistry; for when dentists have reached that stage of their profession when no artificial substitute for natural teeth are required, then will medicine have stamped out all disease and man will have eaten of the tree of perpetual life.

